# Engineering Properties of GeSi Alloy Quantum Dots by High-Temperature Annealing

**DOI:** 10.3390/nano16120736

**Published:** 2026-06-13

**Authors:** Wei Luo, Yang Yin, Qiang Huang, Jingpu Yang, Yan Zhan, Zitong Liu, Zuimin Jiang, Changlin Zheng, Zhenyang Zhong

**Affiliations:** State Key Laboratory of Surface Physics and Department of Physics, Fudan University, Shanghai 200438, China; 22110190039@m.fudan.edu.cn (W.L.); 22110190074@m.fudan.edu.cn (Y.Y.); 21210190026@m.fudan.edu.cn (Q.H.); 22210190046@m.fudan.edu.cn (J.Y.); 21110190065@m.fudan.edu.cn (Y.Z.); 24110190047@m.fudan.edu.cn (Z.L.); zmjiang@fudan.edu.cn (Z.J.)

**Keywords:** GeSi alloy quantum dots, high temperature annealing, 1.55 µm emission, tensile strain

## Abstract

GeSi alloy quantum dots (QDs) are a promising candidate for a light source implemented in Si-based monolithic optoelectronic integrated circuits (MOEICs) thanks to their telecom-wavelength emission and the compatibility with the Si integration technology. Herein, the engineering properties of GeSi alloy QDs are demonstrated via rapid thermal annealing (RTA). The PL spectra of GeSi alloy QDs exhibits remarkably enhanced intensity and an initial red shift followed by a blue shift with increasing annealing temperature. Particularly, it can be characterized as a single narrow peak at ~1.55 µm of the intensity enhanced by ~20 times after the RTA at 1100 °C. These features are attributed to the progressively enhanced intermixing and the abnormal transition from compressive strain to tensile strain in QDs with increasing annealing temperature, which are demonstrated by Raman spectra and transmission electron microscopy (TEM) images. Moreover, a large polycrystalline-domain appears around QD at a sufficiently high annealing temperature. It facilitates the tensile strain in QDs, which arises during the RTA due to the thermal expansion coefficient mismatch between Ge and Si. These results demonstrate that high-temperature annealing can efficiently modulate the properties of GeSi alloy QDs, particularly for emission at 1.55 µm, which may have great potential for an efficient Si-based light source.

## 1. Introduction

The surge of data processing, high-performance computing, and communication demand is driving unprecedented requirements for broadband, high-density, and high-speed on-chip signal interconnections, particularly with the rapid expansion of artificial intelligence (AI) [1]. Among diverse emerging technologies, monolithic optical-electronic integrate circuits (MOEICs) on silicon are a promising key technology for the scalable integration of high-performance photonic components, such as waveguides [2,3], modulators [4,5], detectors [6,7,8,9], and resonators [10], which also enable efficient fiber-to-chip connections [11,12,13]. So far, on-chip light sources based on Si remain a formidable roadblock due to the poor quantum efficiency originating from the indirect bandgap nature of silicon [14,15,16], particularly at a telecom-wavelength of 1.55 µm [17,18,19]. Tremendous efforts have been devoted to overcome this shortage, including silicon nanocrystals [20], erbium doping in silicon [21,22], silicon Raman lasers [23,24] and III-V lasers hybrid-integrated on silicon [25,26,27]. Given the quasi-direct bandgap with the emission at near-infrared optical communication wavelengths, high hole mobility and the compatibility with CMOS processes, Ge exhibits significant promise for applications in MOEICs [28]. To further improve the optical properties of Ge, two primary strategies can be employed, exploiting the quantum confinement effect of Ge or GeSi quantum dots (QDs) to improve quantum efficiency [29], achieving direct-bandgap emissions in highly n-doped/tensile–strain Ge or GeSn alloys by band engineering [30,31]. In addition, thermal annealing plays a pivotal role in engineering the properties of self-assembled GeSi alloy QDs. It can improve crystalline quality via promoting inter-diffusion and defect annihilation, thereby enhancing radiative efficiency by significantly suppressing non-radiative recombination [32,33]. Particularly, it facilitates tuning the strain and the compositions of heterostructures, which can lead to pronounced modulations of energy levels and in turn affect carrier confinement and transition dynamics. For instance, compressive strain in Ge can induce crystal structure transformation from a cubic lattice to a hexagonal lattice via generally termed stacking faults [34,35,36], while tensile strain can decrease the energy difference between the Γ-valley and the L-valley of the conduction band of Ge to promote direct bandgap transitions [37]. The modulations of compositional intermixing and strain allow for the tuning of the emission wavelength, and even for the changing of the localization of carriers to result in type-I or type-II bandgap emission [38]. Accordingly, the comprehensive investigations of the effects of high-temperature annealing on the lattice structure, morphological evolution, composition, strain and the optical properties of GeSi alloy QDs are in high demand.

Herein, we systematically elucidate the impacts of high-temperature annealing on the properties of GeSi alloy QDs. The progressively enhanced photoluminescence (PL) spectra of QDs with an unprecedented red shift is demonstrated as increasing the annealing temperature. Interestingly, the PL spectrum of QDs can be characterized as a single narrow peak at ~1.55 µm with the intensity enhanced by ~20 times for an ultra-high annealing temperature of 1100 °C. These features are attributed to the pronounced intermixing and the abnormal tensile strain around QD after the high-temperature annealing, which are demonstrated by Raman spectra and transmission electron microscopy (TEM). Particularly, the transition from the compressive strain for the lattice mismatch to the tensile strain for the thermal expansion coefficient mismatch between Ge and Si can occur due to the appearance of large polycrystalline-domain around QDs after sufficiently high-temperature annealing. Based on self-consistent calculations, the emissions related to type-I and type-II bandgap are taken into account for the abnormal red shift of PL peak with annealing temperatures. These results reveal that a high-temperature annealing enables a dramatic enhancement of emission at ~1.55 µm from GeSi alloy QDs desired for innovative light source in Si-based MOEICs.

## 2. Materials and Methods

The samples are grown on Si (001) substrates by solid source molecular beam epitaxy in a Riber Eva-32 system. The Si substrates are cleaned by the RCA method and a subsequent HF treatment for a hydrogen-terminated surface. After thermal desorption, a Si buffer layer of 40 nm is grown at a rate of 0.81 Ås^−1^ while ramping the temperature from 500 °C to 600 °C. Then, a stack of five layers of Ge QDs are grown at a rate of 0.25 Ås^−1^ via the Stranski-Krastanov growth mode. For each QD layer, ~2 nm Ge is deposited from 420 °C to 520 °C to reduce alloying at the interface and promote QD formation later. These QD layers are separated by 20 nm Si spacer layers. Finally, a Si cap layer of 80 nm is grown at 450 °C. The rapid thermal annealing (RTA) is performed for samples under different conditions (650 °C for 3 min, 900 °C for 90 s, 1000 °C for 45 s, 1100 °C for 45 s, labeled as sample B, C, D, E, respectively) in the forming gas. The sample without annealing is labeled as sample A. For analyzing surface morphologies of GeSi alloy QDs, a reference sample containing one layer of QD without the cap layer is also grown.

The surface morphologies of GeSi alloy QDs are characterized by atomic force microscopy (AFM) (Veeco DI Multimode V SPM) in tapping mode. Raman spectra are obtained at room temperature in the backscattering configuration using a Jobin Yvon HR-Evolution 2 micro-Raman spectrometer. The excitation source is a 532 nm line of a solid-state laser, which is focused on the sample surface with a spot of ~10 μm in diameter. The high-angle annular dark field (HAADF) scanning transmission electron microscopy (STEM) images are obtained by a double aberration corrected STEM/TEM (Themis Z, Thermo Fisher Scientific) operated at 300 kV. The aberration-free probe semi-convergent angle is set to 21.4 mrad to achieve ultra-high resolution better than 0.1 nm. A Fischione Instruments 3000 ADF detector spanning an angular range from 79 to 200 mrad is used to collect the HAADF images. The cross-sectional electron-transparent TEM foil is prepared by a focused ion beam (FIB) (Thermo Scientific Helios G4 CX). The photoluminescence (PL) measurements are carried out in a closed-cycle helium cryostat whose temperature range is from 20 to 300 K. The PL spectra is analyzed with a monochromator (Omni-λ500, Zolix Instruments Co.) and detected with an extended InGaAs photodetector using the standard lock-in technique. The excitation source is a 532 nm solid-state laser, which is focused on the sample surface with a spot of ~2 mm in diameter.

**Figure 1:**
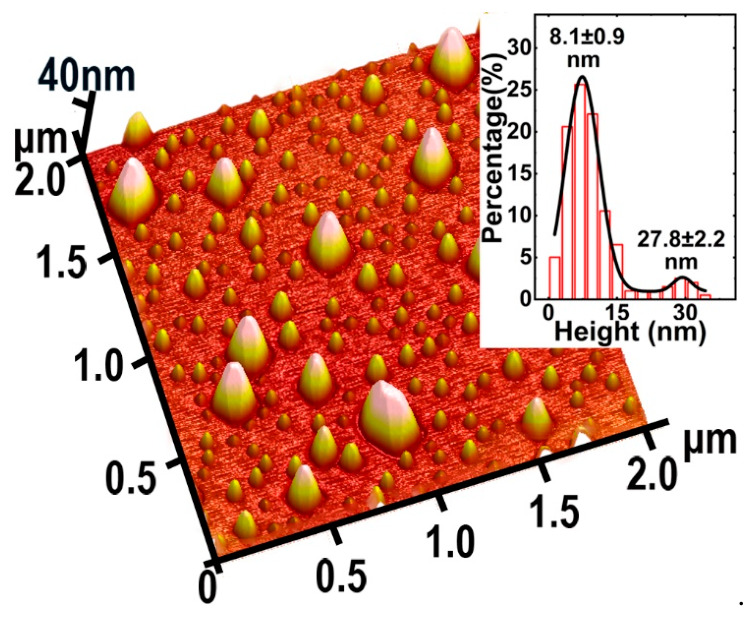
AFM image of GeSi alloy QDs. The inset shows the height distribution of GeSi alloy QDs.

Figure 1 shows the surface morphology of the GeSi alloy QDs on a Si (001). The QDs of two distinct sizes are clearly observed. The small QDs mainly comprise dome-like QDs. The big ones are superdome-like QDs, which are generally formed for sufficient Ge deposition [39]. The inset is the height distribution of these QDs. It can be fitted by two Gaussian peaks, corresponding to the two sizes of QDs. The average heights (±standard deviation) of the small and the big GeSi alloy QDs are 8.1 (±0.9) and 27.8 (±2.2) nm, respectively. In addition, the densities of the small and the big QDs are 5 × 10^9^ cm^−2^ and 4.5 × 10^8^ cm^−2^, respectively. The formation of the big QD is attributed to the coarsening process of the small QDs through Ostwald ripening and in turn the decrease in their areal density [40]. It is noteworthy that the overall volume of GeSi alloy QDs is greater than the nominally deposited volume of Ge. This indicates that some Si atoms are incorporated into QDs during growth to partially relax the misfit strain [41].

## 3. Results

### 3.1. The PL Spectra of the Samples

**Figure 2:**
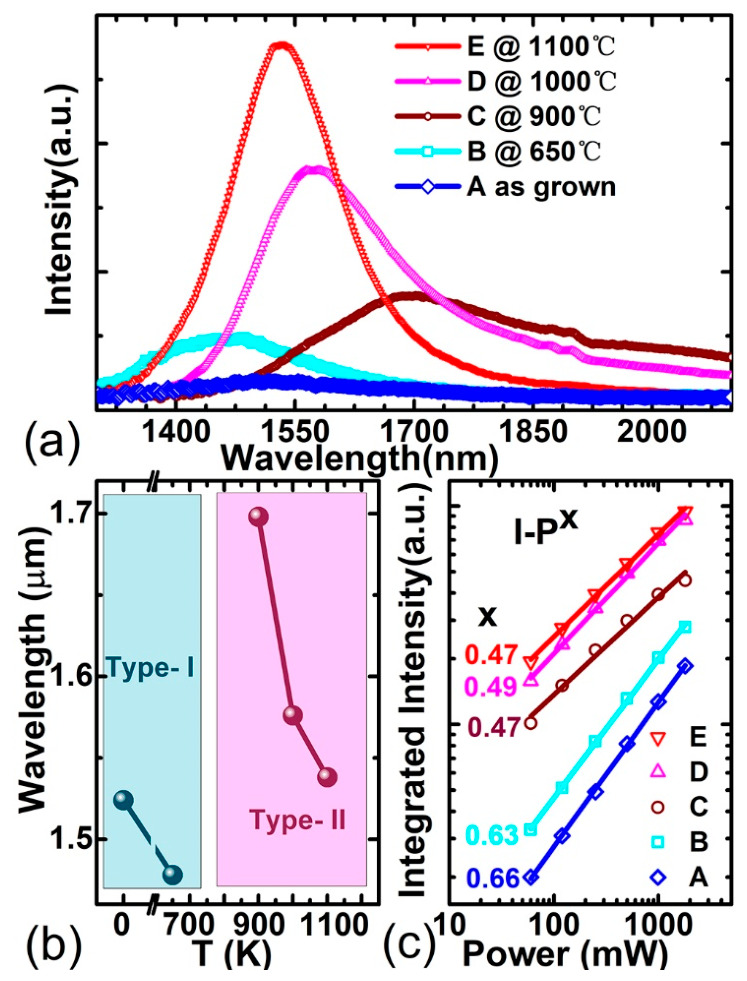
(**a**) PL spectra of the samples A–E for an excitation power of 500 mW at 20 K, (**b**) the wavelength of PL peak vs. annealing temperature, (**c**) the integrated intensity of PL peak vs. excitation power for the samples A–E. The as-grown sample A is considered as annealed at 0 °C.

Figure 2a shows the PL spectra of the samples A–E for an excitation power of 500 mW at 20 K. It can be seen that the PL intensity remarkably increases with the increase in annealing temperature. This means that the high-temperature annealing is a feasible strategy to reduce non-radiative recombination centers in samples. Moreover, for the sufficiently high annealing temperature (e.g., ≥900°), the PL spectrum tends to become narrow when increasing the annealing temperatures, as demonstrated in Table S1 in the Supplementary Materials. At the ultra-high temperature of 1100 °C, the PL spectrum can be characterized as a single narrow peak, as demonstrated in Figure S1 in the Supplementary Materials. Particularly, the intensity of its peak approximately at 1.55 µm is enhanced by 20 times in comparison with that of the as-grown sample. The wavelength of PL peak with annealing is shown in Figure 2b. Obviously, the PL peak blue-shifts for the samples A–B and the samples C–E with the annealing temperature. This is reasonable since the intermixing of Ge/Si is progressively enhanced with increasing the annealing temperature. Therefore, the Ge composition in QDs decreases, leading to a blue shift in the emissions. At an ultra-high annealing temperature, e.g., 1100 °C, the intermixing of Ge/Si can occur throughout the whole QD. Accordingly, the Ge composition and the strain in QDs tend to be uniform, resulting in the narrow PL peak. However, the PL peak red shifts dramatically for the samples B–C with the annealing temperature increased form 650 °C to 900 °C. Moreover, this abnormal red shift of PL peak is accompanied with the considerably different power dependence of PL intensity, as shown in Figure 2c. The integrated PL intensities (I) as a function of excitation power (*p*) can generally be fitted by $I\sim p^{x}$. Surprisingly, the indices *x* can be categorized into two types, ~0.65 for samples A–B and ~0.48 for samples C–E. These results indicate that the emission mechanism of QDs in the samples A–B seems to be different from that in the samples C–E. The unique features of the PL spectra demonstrate the dominant emissions from the GeSi alloy QDs rather than defects. More details are in the Supplementary Materials.

### 3.2. The Raman Spectra of the Samples

**Figure 3:**
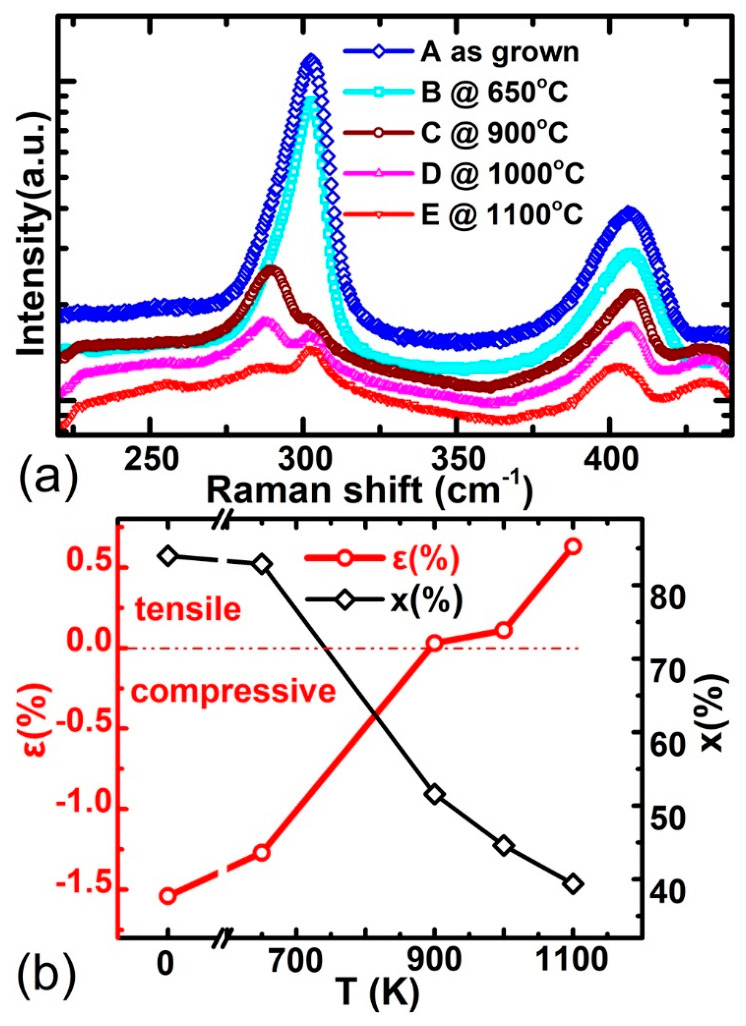
(**a**) Raman spectra of all samples, (**b**) the Ge composition and the strain in QDs vs. the annealing temperature in the samples A–E. The as-grown sample A is considered as annealed at 0 °C.

To clarify those unique properties of the PL spectra, Raman spectra of all samples are measured, as shown in Figure 3a. A peak around ~400 cm^−1^ corresponds to the so-called Si-Ge local modes [42]. For both samples A–B, a Raman peak at ~302 cm^−1^ is observed, arising from the overlap between the Ge–Ge vibrational mode and the Si second-order transverse acoustic (2TA) phonon mode [43]. After RTA at sufficiently high temperatures, these overlapping peaks are separated into two distinct modes at ~287 cm^−1^ and ~302 cm^−1^ for the samples C–E. This demonstrates that the high annealing temperature leads to the red shift in the Ge-Ge mode. Based on the Raman spectra, the Ge composition and the strain in QDs can be estimated by the following formulas [44]

(1) $$\left\{\;\begin{array}{c} \omega^{Si-Si}\left(x,\varepsilon \right)=520.7-66.9x-730\varepsilon \;\\ \omega^{Si-Ge}\left(x,\varepsilon \right)=400.1+24.5x-4.5x^{2}-33.5x^{3}-570\varepsilon \;\\ \omega^{Ge-Ge}\left(x,\varepsilon \right)=280.3+19.4x-450\varepsilon \; \end{array}\right.$$

where *ω*, *x* and *ε* are the measured Raman mode frequency, the Ge composition and the elastic strain, respectively. The Ge composition and the strain in QDs under different RTA conditions are shown in Figure 3b. It can be seen that the average Ge composition x in QDs progressively decreases from ~84.0% to ~39.4% with an increase in annealing temperature. This originates from the intermixing of Ge/Si during RTA. The higher the annealing temperature, the more intermixing of Ge/Si there is, and in turn leads to less Ge composition in QDs. Particularly, for the sufficiently high annealing temperatures, the elastic strain ε transitions from compressive (e.g., −1.27% @ 650 °C) to tensile (e.g., +0.63% @ 1100 °C) strain. Given the larger lattice constant of Ge, the compressive strain generally exists in self-assembled GeSi alloy QDs. The strain transition cannot be induced by the intermixing of Ge/Si. This means that annealing at sufficiently high temperature may generate large structural deformation around QDs to facilitate complete relaxation of the compressive strain in QD. Accordingly, GeSi alloy QDs within Si can have little or no strain at sufficiently high temperatures during annealing, but develop tensile strain upon the subsequently rapid cooling during RTA due to the mismatch in thermal expansion coefficient of Si and Ge [45]. This provokes a shift in Ge-Ge mode to lower wavenumbers. It should be noted that the strain and composition values derived from the Raman spectra correspond to spatially averaged quantities over the laser spot area, which contains a large number of GeSi QDs and surrounding matrix material. To obtain the exact distributions of the strain and the composition around a GeSi QD, systematic analyses of high-resolution TEM images are necessary. The tensile strain generated in this process is promising to facilitate the transition of QDs to be a direct bandgap material.

### 3.3. The Dramatic Morphological Transition of the Sample B and D

**Figure 4:**
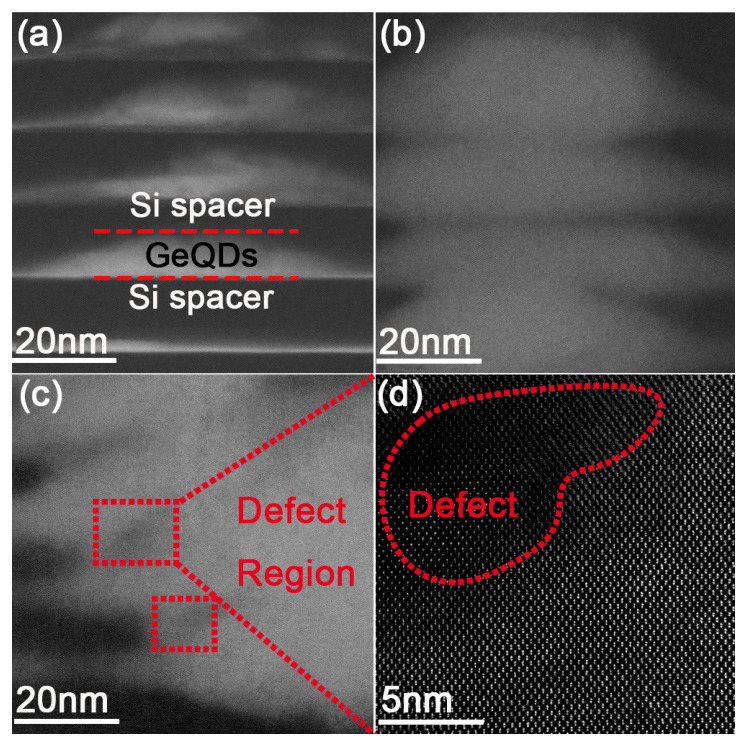
HAADF-STEM images of stacked QDs in (**a**) the sample B and (**b**) the sample D, (**c**) STEM image of defect regions (in dashed boxes) around QDs in the sample D, (**d**) the high-resolution STEM image of the defect region denoted in (**c**).

In order to further elucidate the impact of high-temperature annealing on the structure and optical properties of QDs, the HAADF-STEM images of the sample B and D are shown in Figure 4. They reveal a dramatic morphological transition. For sample B, coherent vertical-correlated GeSi alloy QDs are spatially isolated by the Si spacer layers with sharp interfaces, as shown in Figure 4a. In contrast, the vertically aligned QDs can be nearly coalescent in sample D, as shown in Figure 4b. This demonstrates that the high-temperature annealing indeed results in the pronounced intermixing of Ge/Si to smear the interface between QDs and the surrounded Si matrix. This annealing-enhanced intermixing of Ge/Si is consistent with the Raman spectra that demonstrate the decrease in Ge composition in QDs with annealing temperature. Furthermore, the high-resolution STEM image identifies some large regions of polycrystalline defects around the QDs, which are highlighted by the red dashed boxes in Figure 4c. The single crystal structure in those regions are significantly modified, as demonstrated by the blurred atomic configuration in the red dashed circle in Figure 4d. Such large polycrystalline domains facilitate the transition from the general compressive strain for the lattice mismatch to the abnormal tensile strain for the thermal expansion coefficient mismatch. These observations validate the above analysis of strain transition with annealing temperature from Raman spectra. On the other hand, this defect of polycrystalline domain does not seriously degrade the optoelectronic properties of QDs since a rather strong PL is observed, as shown in Figure 2.

### 3.4. The Diagram of Conduction Band (CB) Minimum and the Valence Band (VB) Maximum

**Figure 5:**
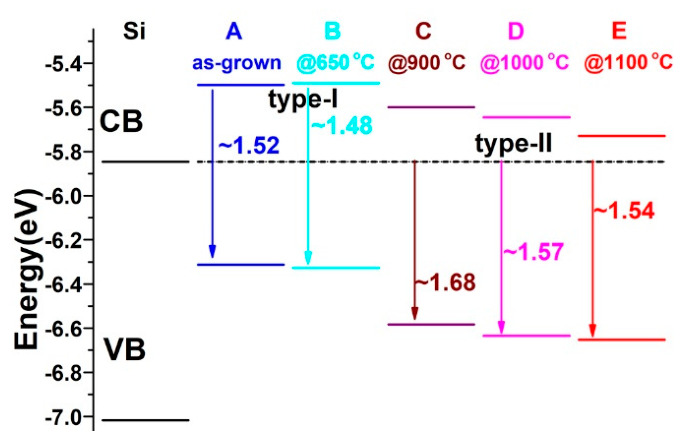
The diagram of conduction band (CB) minimum and the valence band (VB) maximum of all samples and the Si via self-consistent calculations. The emission wavelengths (unit: µm) related to the type-I (for the samples A–B) and the type-II bandgap (for the samples C–E) are shown and denoted by arrows.

Based on the Ge composition and the strain in QDs from the Raman spectra, the band structures of samples A–E are estimated via self-consistent calculations [46]. The conduction band (CB) minimum and the valence band (VB) maximum of all samples in comparison with those of Si are shown in Figure 5. For the samples A and B, the calculated energies of the bandgap corresponding to the type-II band alignment, i.e., the holes confined in QDs and the electrons located in the Si region around the QDs and termed as type-II bandgap, are below 0.5 eV. They are substantially different from the PL peaks in Figure 2. The related emission cannot be observed by the present detector, whereas the calculated energies of the bandgap for both electrons and holes in the QDs, termed as type-I bandgap, give rise to the emission wavelength of 1.52 and 1.48 µm, respectively, as shown in Figure 5. They agree well with the PL peaks in Figure 2. The high Ge composition and the small QDs due to the weak intermixing of Ge/Si for samples A and B result in the well confined holes, which may attract some electrons into QDs due to the rather strong Coulomb interactions. This scenario accounts for the PL peak of rather short wavelength corresponding to the type-I bandgap for the samples A and B, as shown in Figure 2. It also contributes to the weak PL intensity for the samples A and B. In contrast, the calculated energies of the type-II bandgap can lead to the emission wavelength of 1.68, 1.57 and 1.54 µm for the sample C, D, and E, respectively. They are consistent with the PL peaks in Figure 2. Such different emissions related to type-I and type-II bandgap account for the abnormal red shift of PL peaks of QDs in the samples C–E with respect to those in the samples A–B while increasing the annealing temperature in Figure 2, given the different location of electrons for the recombination. Moreover, the excitons around QDs for the type-II bandgap with the separation of electrons and holes in the real space seems to be less favorable for the recombination in the samples C–E with respect to those for the type-I bandgap in the samples A–B. Accordingly, the power indices (~0.48) of the integrated intensity of PL peaks of samples C–E are smaller than those (~0.65) of samples A–B, as shown in Figure 2c. This is consistent with the previous results [47]. It is worth mentioning that the valence band offset between the GeSi alloy QD and Si in sample E is ~0.36 eV. Accordingly, the general three-dimensional (3D) confinement of holes in the QDs persists, although the discrete quantum confinement effect becomes rather weak for some merged QDs at the scale of ~100 nm. Such a 3D confinement can suppress the diffusion of carrier to defects and in turn efficiently reduce the probability of non-radiative recombination during the carrier migration, thereby enhancing the PL intensity. In addition, the annealing temperature of 900–1100 °C is too high for general CMOS backend processes. This issue can be resolved by localized annealing techniques, e.g., femtosecond laser annealing [48]. The femtosecond laser irradiation can result in highly localized and ultrafast heating, which enables the required intermixing and strain engineering within selected regions while minimizing thermal diffusion to surrounding areas [48]. In a practical device process, such localized annealing can be implemented after patterning for devices, where the laser is selectively scanned or focused onto predefined active regions without affecting adjacent structures. Such a local annealing comparable to the high-temperature RTA can potentially achieve the optical benefits described above without significantly affecting adjacent structures or exceeding the thermal budget compatible with CMOS technology.

## 4. Discussion

In summary, the effects of high-temperature annealing on the optical and structural properties of GeSi alloy QDs grown on Si (001) substrates are systematically investigated. Remarkably enhanced PL spectra with an unprecedented red shift are observed by increasing the annealing temperature. Particularly, a sharp peak at telecom C-band of ~1.55 µm accompanied by an enhancement in intensity of ~20 times is realized by RTA at 1100 °C. The PL enhancement is associated with a reduction in non-radiative recombination due to the RTA. Future time-resolved PL studies will be valuable for quantifying carrier lifetime and clarifying the relative contributions of radiative and non-radiative recombination processes. The evolution of Ge composition and the abnormal transition from the compressive strain to the tensile strain in QDs with increasing annealing temperature are demonstrated by Raman spectra. They are attributed to the RTA-induced intermixing of Ge/Si and the appearance of a large polycrystalline region around QDs at sufficiently high annealing temperatures, which are corroborated by the STEM image. The band analyses based on the self-consistent calculations unveil the emissions related to the type-I and type-II bandgap with different recombination rates, which account for the abnormal red shift of PL peak with annealing temperatures. These results indicate that the high-temperature annealing can substantially modulate the properties of GeSi alloy QDs, particularly for emissions at ~1.55 µm, which is promising for a Si-based innovative optoelectronic device compatible with CMOS technology.

## Data Availability

The original contributions presented in this study are included in the article/Supplementary Materials. Further inquiries can be directed to the corresponding authors.

## Supplementary Materials

The following supporting information can be downloaded at: https://www.mdpi.com/article/10.3390/nano16120736/s1. Table S1: The FWHM of PL spectra of samples A–E; Figure S1: The PL spectra of sample E for the excitation power of 500 mW at 20K (blue circle) and a single Gaussian fitting (red line); Figure S2: (a) Temperature-dependent PL spectra of sample E for the excitation power of 500 mW, (b) the peak wavelength as a function of temperature for sample E.
