# Supplementary material for: Engineering Properties of GeSi Alloy Quantum Dots by High-Temperature Annealing
Source: Nanomaterials (Basel). 2026 Jun 13;16(12):736. doi: 10.3390/nano16120736 (PMC13304952; doi:10.3390/nano16120736)
Supplement: Supplementary file 1 [file nanomaterials-16-00736-s001.zip › nanomaterials-4343026-supplementary.pdf]

Supplementary Materials

for the article W. Luo et. al. " Engineering Properties of GeSi alloy Quantum Dots by High-Temperature Annealing"

S1. The single narrow PL peak of sample E

At the lower annealing temperatures, the photoluminescence (PL) spectra exhibit very broad peaks across a wide spectral range, as demonstrated in Table S1, which indicates the coexistence of multiple recombination channels. The spectral width tends to decrease with increasing annealing temperature for the sufficiently high annealing temperature (e.g. ⩾900°). For the sample E (annealing at 1100 °C), the PL peak can be well fitted by a single Gaussian function, as shown in Fig. S1. Although the full width at half maximum (FWHM) is slightly large, the PL peak is the narrowest in comparison with those of other samples.

Table S1. The FWHM of PL spectra of samples A-E.

Sample	FWHM (nm)
A	330
B	240
C	440
D	222
E	136

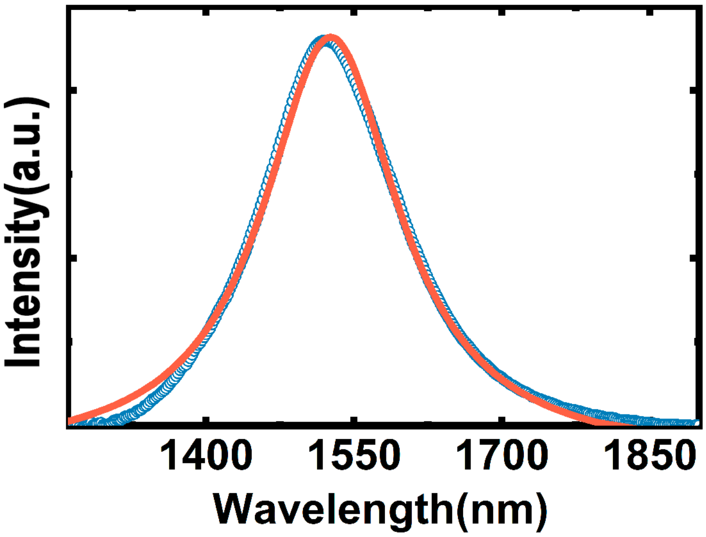

Figure S1. The PL spectra of sample E for the excitation power of 500 mW at 20K (blue circle) and a single Gaussian fitting (red line).

S2. The distinguished features of the PL spectra of samples from the emission of defects.

The temperature dependent PL spectra of sample E is shown in Fig. S2(a). The wavelength of PL peak as a function of temperature is shown in Fig. S2(b). Obviously, the PL peak of sample E considerably shifts with

increasing temperature. This is similar to previous result [1]. Moreover, the PL peak can be distinguished even at room temperature, although it becomes weak with increasing temperature. In general, the defect-related PL peaks are rather narrow and quickly quench with temperature [2, 3]. It can hardly be observed above 100K. For the defect-related PL peaks at ~1550 nm, it slightly redshift with increasing temperature [2]. These features of defect-related PL peaks are remarkably different from those of the PL peak of sample E. In addition, the wavelengths of defect-related PL peaks are essentially not affected by the annealing temperature. Moreover, the intensities of PL peaks of samples become stronger with the increase of annealing temperature. This is consistent with the previous results since the high-temperature annealing can improve crystalline quality via promoting inter-diffusion and defect annihilation, thereby enhancing radiative efficiency by significantly suppressing non-radiative recombination [4, 5]. Accordingly, the observed PL peak of sample E, as well as other samples, essentially originates from GeSi alloy QDs rather than from defects.

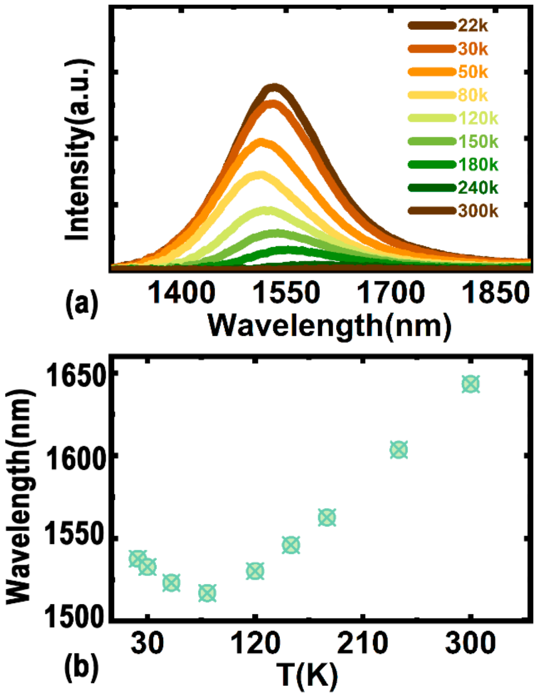

Figure S2. (a) Temperature-dependent PL spectra of sample E for the excitation power of 500 mW, (b) the peak wavelength as a function of temperature for sample E.

REFERENCES

[1] C. Lee, Y. Yoo, B. Ki, M. Jang, S. Lim, H. Song, J. Cho, J. Oh and Y. Cho, Sci Rep **9**, 11709 (2019).

[2] H. Lee, and S. Choi, J. Appl. Phys. **85**, 1771 (1999).

[3] Y. Baron, A. Durand, P. Udvarhelyi, T. Herzig, M. Khoury, S. Pezzagna, J. Meijer, I. Robert-Philip, M. Abbarchi, J.-M. Hartmann, V. Mazzocchi, J.-M. Gérard, A. Gali, V. Jacques, G. Cassabois, and A. Dréau, ACS Photonics **9**, 2337 (2022).

[4] Q. Cai, H. Zhou, F. Lu, Appl. Surf. Sci. **253,** 4792 (2007).

[5] S. Fukatsu, N. Usami, and Y. Shiraki, J. Vac. Sci. Technol. B **11**, 895 (1993).
